# The Antipyretic Effect of High-Dose Paracetamol Versus Mefenamic Acid in the Treatment of Febrile Children: A Randomized Control Trial

**DOI:** 10.7759/cureus.26733

**Published:** 2022-07-11

**Authors:** Amruta Loya, Mohd Saeed Siddiqui, Avinash Sangle, Vinod Ingale, Shreya Saha, Madhurasree Nelanuthala

**Affiliations:** 1 Department of Pediatrics, Mahatma Gandhi Mission (MGM) Medical College and Hospital, a Constituent Unit of MGM Institute of Health Sciences, Aurangabad, IND

**Keywords:** high-dose paracetamol (20 mg/kg), high-dose acetaminophen, antipyretic effect, mefenamic acid, febrile children

## Abstract

Introduction

Fever is the most common presenting symptom in children and causes distress in patients and parents. Although nonsteroidal anti-inflammatory drugs (NSAIDs) are commonly used as antipyretics, they should be reserved for pain or chronic inflammatory conditions due to safety concerns. If we can safely achieve the same antipyretic effect using a higher dose (20 mg/kg) of paracetamol, NSAIDs may be avoided for treating fever. There is a paucity of literature comparing the antipyretic effect of mefenamic acid and high-dose paracetamol. We hypothesized that there would be no difference in the antipyretic effect of high-dose paracetamol and mefenamic acid.

Methods

In this randomized control trial, 165 febrile children were randomly allocated to one of the following three groups: standard-dose (15 mg/kg) paracetamol (SDPCM) as the control group and high-dose (20 mg/kg) paracetamol (HDPCM) and mefenamic acid (6 mg/kg) (MFN) as the intervention groups. The temperature was measured using a digital thermometer at the start of drug dosage and every 15 minutes until it reached normal. One-way between-group analysis of variance (ANOVA) was used to compare outcome measures such as time for temperature to reach normal, fall of temperature in 60 minutes, and time for the next fever. Post hoc analysis was performed to compare mean differences. Patients were monitored for adverse effects.

Results

Out of 165 enrolled patients, 159 were analyzed. The baseline demographic data were comparable among the groups. There was a statistically significant difference in the mean time taken for the temperature to reach normal (F-value (F) (2,156)=3.184, p<0.05) and the mean reduction in temperature at 60 minutes (F (2,156)=23.40, p<0.001) among the groups. The mean time for temperature to reach normal in the SDPCM group (97.50±26.60 minutes) was longer than that in the HDPCM (85.09±31.43 minutes) and MFN (84.90±30.42 minutes) groups. The decrease in temperature over 60 minutes was greater in the HDPCM (0.46°C±0.19°C) and MFN (0.45°C±0.11°C) groups than in the SDPCM (0.33°C±0.10°C) group. The time to the next fever spike was shorter for the SDPCM group (5.07±2.66 hours) than for the HDPCM (7.20±3.08 hours) and MFN (8.82±3.83 hours) groups. Post hoc analysis demonstrated that high-dose paracetamol and mefenamic acid had similar and faster antipyretic effects than standard-dose paracetamol. Although the duration of action was found to be longer in the mefenamic acid group, the difference was not statistically significant. There were negligible adverse effects in the groups.

Conclusion

Standard-dose paracetamol (15 mg/kg/dose) had a slower and shorter antipyretic effect than high-dose paracetamol (20 mg/kg/dose) and mefenamic acid (6 mg/kg/dose). A single dose of high-dose paracetamol was safe and had a similar antipyretic effect as mefenamic acid. Mefenamic acid may be avoided as an antipyretic and spared for pain and anti-inflammatory indications. Multicentered double-blind clinical trials with larger sample sizes and comparisons of other NSAIDs will be required to confirm these findings.

## Introduction

Fever is a common presentation in children in various conditions. It is a helpful inflammatory protective response and beneficial host defense. Antipyretics should not be used for bringing body temperature to normal but to relieve the discomfort of the child [1], high-grade fever with anticipated febrile convulsion, and inflammatory conditions or for mild analgesia. There are many misconceptions among parents and healthcare providers about the nature of fever, the use of antipyretics, doses, and the frequency of their use in contrast to accumulating evidence [2]. Antipyretics are often used as monotherapy, combined or alternating, and some evidence favors the use of alternating antipyretic agents [3].

Although nonsteroidal anti-inflammatory drugs (NSAIDs) should be reserved for pain and chronic inflammation, they are frequently used for their antipyretic effect. Ibuprofen is found to be safe in children above six months, but concerns have been raised in children with comorbidities [4]. Children with diarrhea and volume depletion or preexisting chronic renal failure who received ibuprofen for fever were reported to develop acute renal failure in a case series [5].

Mefenamic acid is also used as an effective antipyretic and is well tolerated by children. Mefenamic acid had better antipyretic activity than paracetamol (10 mg/kg), but its antipyretic activity continued well beyond four hours [6].

Paracetamol is safe and recommended as the antipyretic of first choice [4]. It is also used safely in higher doses for a short duration. Even a single dose of 30 mg/kg at bedtime was shown to increase the sleep time of the whole family, especially in mild infection, but it is cautioned not to use a high dose of paracetamol repeatedly. A dose of 20 mg/kg is found to cause a larger and more prolonged decrease in temperature [7]. Additionally, single-dose rectal acetaminophen provided effective postoperative analgesia for children who underwent ophthalmic surgery at high (40 mg/kg) and low (20 mg/kg) doses in early postoperative use as well as over a 24-hour period [8]. Hepatoxicity with paracetamol is very rare in children if it is used in the therapeutic range (≤75 mg/kg per day orally or intravenously or ≤100 mg/kg per day rectally) [9].

Although NSAIDs are commonly used as antipyretics, they should be reserved for pain or chronic inflammatory conditions due to safety concerns. They are often used due to perceived fast and prolonged action and with parental anxiety and demand for rapid recovery, especially if fever is high grade and continues for more days. If we can safely achieve the same antipyretic effect using a higher dose (20 mg/kg) of paracetamol, other NSAIDs may be avoided for treating fever, as paracetamol is a safe drug of first choice and can even be used in infants above two months. There is a paucity of literature comparing the antipyretic effect of mefenamic acid and high-dose (20 mg/kg) paracetamol.

We hypothesize that there is no significant difference between the antipyretic effect of a single dose of high-dose (20 mg/kg) paracetamol and mefenamic acid. Therefore, this randomized control trial was undertaken to compare the antipyretic effect of mefenamic acid with paracetamol in febrile children.

## Materials and methods

Study design

To compare the antipyretic effect of paracetamol and mefenamic acid, we conducted a parallel, single-blind, multiple-arm, randomized control trial. It was conducted in the department of pediatrics in a tertiary care hospital in the Marathwada region, Maharashtra. The participants were enrolled between October 2017 and October 2019.

Study participants

Children between six months and five years of age of either sex and with an axillary temperature of 38°C or above admitted to the pediatric ward were included in the study.

We excluded the following patients: those who received any antipyretic drug in the previous six hours; those who received antibiotics prior to admission; those receiving warfarin, heparin, or any other antihypertensives; those with a history of hypersensitivity to study medications; those requiring admission in the intensive care unit; patients with active gastrointestinal bleeding and any known coagulopathy; patients with chronic renal, liver, and cardiac failure; and patients with febrile convulsions during the study. Any clinical adverse event, serious illness, or other medical condition (body temperature increasing above 40°C or decreasing below 35.83°C and the occurrence of any severe physical event or convulsion), in which continued participation was not in the best interest of the subject, was planned to be excluded from the study.

Randomization procedure

**Figure 1:**
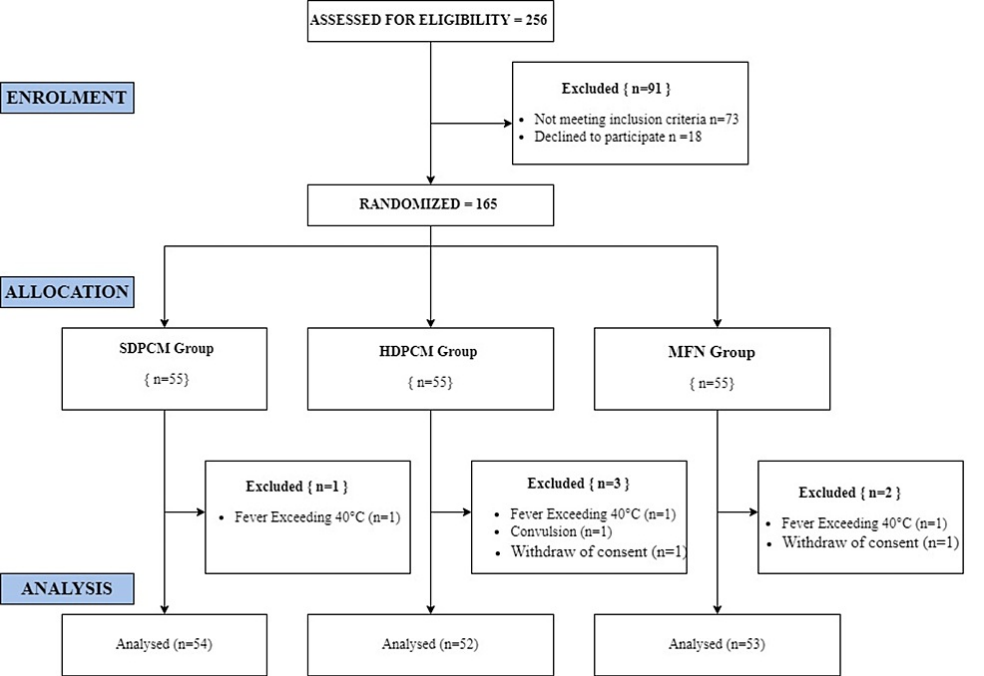
Flowchart showing recruitment of participants

A simple randomization technique was used by drawing chits to allocate participants into the three groups in a 1:1:1 proportion (55 in each group): standard-dose paracetamol (SDPCM), high-dose paracetamol (HDPCM), and mefenamic acid (MFN). As the drugs were not alike and doses were different, the nurse administering the drug and participants could not be blinded, but the statistician analyzing the data was blinded. The recruitment of participants is shown in Figure 1.

Study intervention

Children admitted to the pediatric ward with fever were screened for eligibility. Those meeting the criteria and giving consent were enrolled in the study. Demographic data were collected and entered into a case record form. The participants were randomly allocated to one of the three groups. Pacimol 250 mg/5 mL (Ipca Laboratories, Maharashtra, India) 60 mL bottles were used for the paracetamol groups. Meftal P 100 mg/5 mL (Blue Cross, Maharashtra, India) 60 mL bottles were used for the mefenamic acid group. The bottles of the drug were kept at room temperature. Only the investigator had access to the study drugs.

The participants in the SDPCM group received a standard dose (15 mg/kg) of paracetamol, the HDPCM group received a high dose (20 mg/kg) of paracetamol, and the MFN group received 6 mg/kg of mefenamic acid. Both drugs were administered by a trained staff nurse on duty as a single oral dose after calculating the dose under the supervision of the investigator. Spillage of drug or vomiting during drug administration was observed and compensated by readministering the judged amount. Antibiotics were not used during this period of antipyretic treatment for 12 hours.

Weight (kg) was measured using a calibrated digital weighing machine. The baseline axillary temperature on the degree Celsius (°C) scale was measured at the start of the fever spike in the ward using a calibrated digital thermometer (AGARO DT-555, AGARO, Kolkata, India) by the trained staff nurse on duty. The axillary temperature was recorded before drug administration and then every 15 minutes until it was reduced to normal (<37.22°C).

Study outcomes

Study outcomes were measured for antipyretic effects and the safety and tolerability of drugs. Axillary temperature was measured with a digital thermometer before drug administration and every 15 minutes after the first dose. A temperature below 37.22°C was considered the endpoint. The time (minutes) for the temperature to reach normal (37.22°C) was recorded. A decrease in temperature (°C) in the first 60 minutes was noted. The time of the next fever spike (hours) was also noted to determine the duration of the antipyretic effect.

To monitor the safety and tolerability of the drugs, all the enrolled children were observed clinically for vomiting, dislikeness to meals, daytime sleep, and the need for additional medication. The participants were also observed for withdrawal from the study due to body temperature increasing above 40°C or decreasing below 35.83°C and the occurrence of any severe physical event.

Sample size

The sample size calculated was 159, 53 in each group, for comparing the means of the three groups with a 95% level of confidence, 80% power, and an effect size of 0.25 using the G*Power software version 3.1 (Universität Kiel, Germany) [10]. We enrolled 165 febrile children to make up for potential exclusion.

Statistical analysis

All the data were compiled in an Excel spreadsheet (Microsoft Corporation, Redmond, WA, USA), and a master chart was prepared. For data analysis, IBM SPSS Statistics for Windows version 20.0 (IBM Corp., Armonk, NY, USA) was used. Demographic data were summarized in the form of frequencies and percentages for qualitative data and means and standard deviations for quantitative data. A one-way between-group analysis of variance (ANOVA) test was used to compare the time for temperature to reach below 37.22°C, the fall of temperature in 60 minutes, and the time of the next fever spike. As the ANOVA test uses F statistics, in the article when we reported F (2,156), F refers to F-value, and 2 and 156 refer to degrees of freedom between and within groups, respectively. The chi-square test was used to compare qualitative data. To determine the effect size, eta-squared values were calculated. Tukey’s honestly significant difference (HSD) test was used for post hoc analysis.

Ethical consideration

The study was approved by the ethical and research committee of Mahatma Gandhi Mission (MGM) Medical College and Hospital, Aurangabad, Maharashtra (MGM-ECRHS/2017/51). The study was also registered in the Clinical Trials Registry of India (registration number CTRI/2018/12/016798). Informed consent was obtained from the parents of the patients. Participation was voluntary, and parents had a choice to withdraw from the study at any time.

## Results

In this randomized control trial, we allocated febrile children of six months to five years of age into three groups, as after five years the weight increases and doses in mg/kg may not be appropriate. The SDPCM group received standard-dose paracetamol (15 mg/kg), the HDPCM group received high-dose paracetamol (20 mg/kg), and the MFN group received mefenamic acid (6 mg/kg). As some cases were excluded after allocation (Figure 1), we analyzed 54 in the SDPCM group, 53 in the HDPCM group, and 53 in the MFN group. Baseline demographic data were collected, including age, sex, weight, height, diagnosis, and comparison. The temperature was recorded every 15 minutes in degrees Fahrenheit until the temperature reached baseline, as the patients received a single dose of the drug according to the allocated group. The mean time taken to reach normal and the mean temperature reduction in 60 minutes were compared among the groups and analyzed using one-way between-group ANOVA. The patients were followed for the development of the next fever spike, and the time in hours was compared. The children were also observed for adverse effects, such as vomiting, dislikeness for medicine, and daytime sleepiness, and were compared.

As seen in Table 1, most patients were from the age group of 1-3 years and males in all the groups. They were comparable with respect to mean age (F (2,156)=1.18, p=0.742), sex (χ2(2)=1.24, p=0.410), mean weight (F (2,156)=0.215, p=0.807), mean height (F (2,156)=2.17, p=0.117), and peak temperature (F (2.156)=0.134, p=0.875), with no statistically significant difference among the groups.

**Table 1:** Baseline characteristics and summary statistics of the groups n: number of patients, SD: standard deviation, SDPCM: standard-dose paracetamol, HDPCM: high-dose paracetamol, MFN: mefenamic acid

Parameter		SDPCM group (n=54)	HDPCM group (n=52)	MFN group (n=53)
Sex	Male (n (%))	29 (53.7)	35 (67.3)	40 (75.5)
	Female (n (%))	25 (46.3)	17 (32.7)	13 (24.5)
Age group	<1 year (n (%))	10 (18.9)	13 (25)	13 (24.5)
	1-3 year (n (%))	30 (54.7)	21 (40.4)	23 (43.4)
	3-5 year (n (%))	14 (26.4)	18 (34.6)	17 (32.1)
Age (years) (mean (SD))		3.78 (1.83)	3.97 (1.76)	3.92 (1.83)
Weight (kg) (mean (SD))		10.87 (3.87)	11.26 (3.65)	11.30 (3.79)
Height (cm) (mean (SD))		81.26 (10.81)	77.04 (20.79)	83 (11.72)
Peak temperature (°C) (mean (SD))		38.57 (0.41)	38.56 (0.40)	38.60 (0.43)
Time for temperature to reach normal (minutes) (mean (SD))		97.50 (26.66)	85.09 (31.53)	84.90 (30.42)
Fall of temperature in 60 minutes (°C) (mean (SD))		0.33 (0.10)	0.46 (0.19)	0.45 (0.11)
Return of next fever spike (hours) (mean (SD))		5.07 (2.66)	7.20 (3.08)	8.82 (3.83)

In the SDPCM group, a maximum of 18 (33.9%) patients had viral fever. Additionally, both the HDPCM and MFN groups most commonly had (12 (22.6%) each) viral fever. Other common causes of fever in the study were viral bronchopneumonia, bronchiolitis, and acute gastroenteritis with some dehydration, dengue fever, and acute viral hepatitis. Peak temperature (38.5°C) was also comparable among the three groups (F (2,156)=0.134, p=0.875). A comparison of the mean time for temperature to reach normal (minutes), the mean fall of temperature in 60 minutes (°C), and the mean time for the next fever spike (hours) is also shown in Table 1.

A one-way between-group ANOVA was conducted to explore the antipyretic effect of standard-dose paracetamol, high-dose paracetamol, and mefenamic acid by comparing the time for temperature to reach normal, the fall of temperature in 60 minutes, and the time for the next fever spike (Table 2).

**Table 2:** One-way ANOVA results with effect size η2: eta-squared critical value 0.01 suggests low, 0.06 medium, and 0.14 high effect size; df: degree of freedom Note: *p<0.05: significant; **p<0.001: highly significant

Parameter		Sum of squares	df	Mean square	F value	p value	η^2^
Time to reach normal (minutes)	Between groups	5572.886	2	2786.443	3.184	<0.044*	0.03
	Within groups	136536.548	156	875.234			
	Total	142109.434	158				
Fall of temperature in 60 minutes (°C)	Between groups	0.517	2	0.258	23.407	<0.001**	0.23
	Within groups	1.722	156	0.011			
	Total	2.238	158				
Time of next fever spike (hours)	Between groups	325.912	2	162.956	15.663	<0.001**	0.19
	Within groups	1362.948	131	10.404			
	Total	1688.860	133				

**Figure 2:**
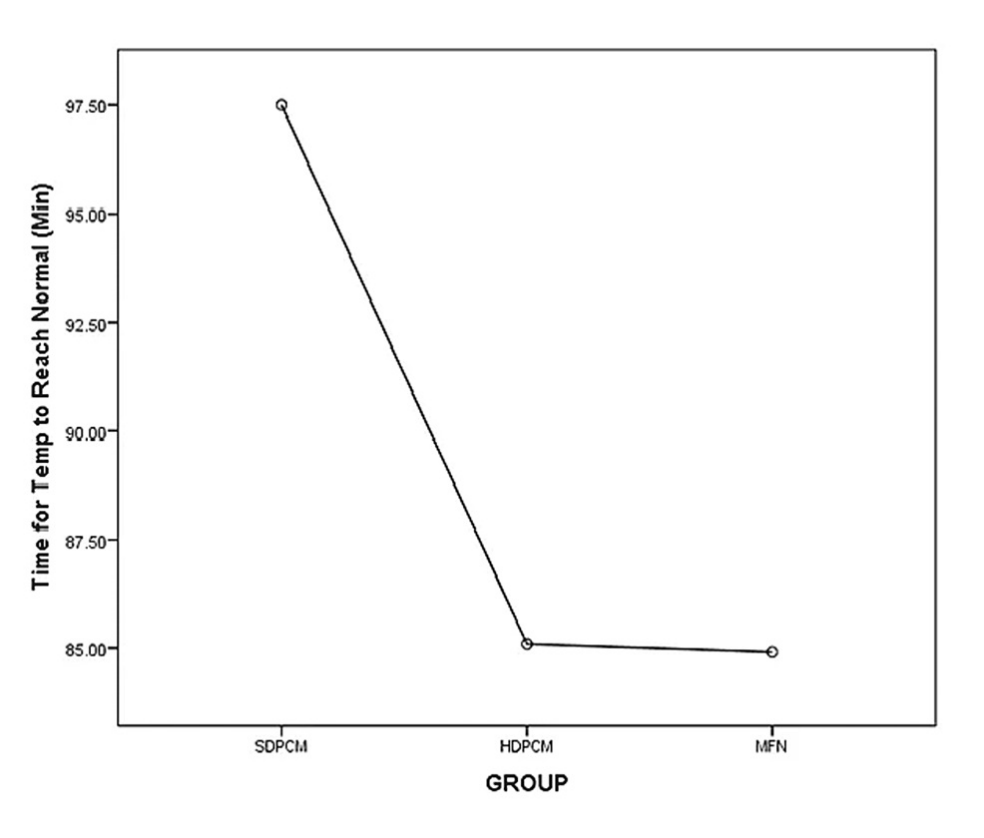
Mean plot showing the time to reach normal temperature

There was a statistically significant difference in the time for temperature to reach normal (F (2,156)=3.184, p=0.044). The effect size, calculated using eta-squared values, was small (η2=0.03). The mean time of the SDPCM group (97.50 minutes) was longer than that of the HDPCM (85.09 minutes) and MFN (84.90 minutes) groups. Post hoc comparisons using the Tukey HSD test indicated that the mean time for temperature to reach normal of the HDPCM group (M=85.09, SD=31.53) did not differ significantly from the MFN group (M=84.90, SD=30.42) (p=0.99) (95% CI: -13.85, 13.47). The mean time for temperature to reach normal was statistically different for the HDPCM (85.09±31.53, p=0.03) and MFN (84.90±30.42, p=0.02) groups as compared to the SDPCM group (97.50±26.65). The mean plot comparing the means of the three groups is shown in Figure 2.

**Figure 3:**
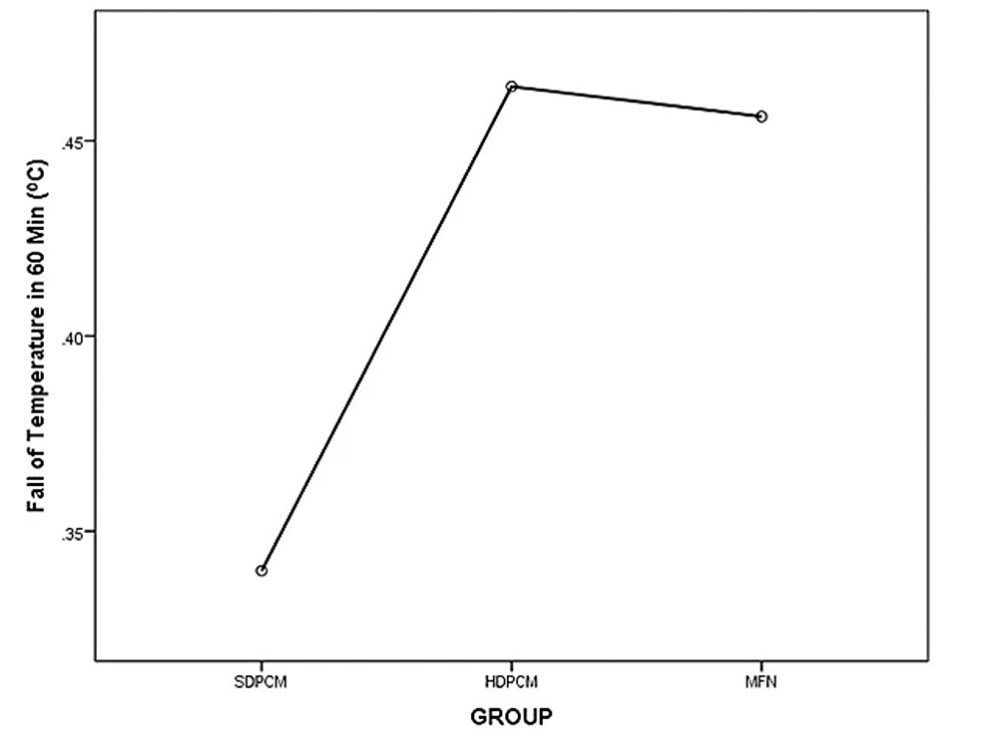
Mean plot showing a decrease in temperature over 60 minutes

There was a statistically significant difference in the decrease in temperature at 60 minutes (F (2,156)=23.40, p<0.001). The effect size, calculated using eta-squared values, was high (η2=0.23). It was greater in the HDPCM (0.46°C) and MFN (0.45°C) groups than in the SDPCM group (0.33°C). Post hoc comparisons using the Tukey HSD test indicated that the mean decrease in temperature after 60 minutes in the HDPCM group (M=0.46, SD=0.19) did not differ significantly from that in the MFN group (M=0.45, SD=0.11) (p=0.925) (95% CI: -0.11, 0.15). The reduction of temperature in 60 minutes was statistically different for the HDPCM (0.46±0.19, p<0.001) and MFN (0.45±0.11, p<0.001) groups as compared to the SDPCM group (0.33±0.10). The mean plot comparing the means of the three groups is shown in Figure 3.

**Figure 4:**
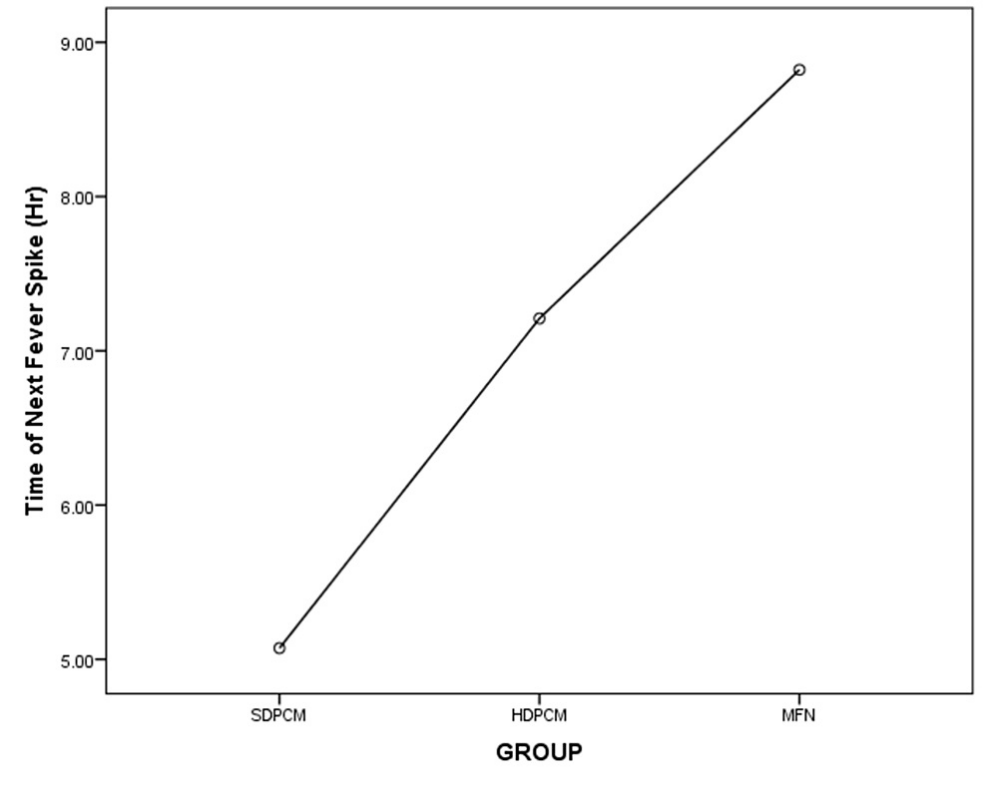
Mean plot showing the time until the next fever spike

Similarly, there was a highly significant difference in the time until the next fever spike (F (2,131)=15.66, p<0.001). The effect size, calculated using eta-squared values, was high (η2=0.19). The time to the next fever spike was shorter for the SDPCM group (5.07 hours) than for the HDPCM (7.20 hours) and MFN (8.82 hours) groups. Post hoc comparisons using the Tukey HSD test indicated that the mean time to the next fever spike in the HDPCM group (M=7.20, SD=3.08) did not differ significantly from that in the MFN group (M=8.82, SD=3.83) (p=0.055) (95% CI: -3.25, 0.02). However, the mean difference of 1.62 hours is practically significant and suggests a longer duration of the antipyretic effect of mefenamic acid. The time for the next fever spike was statistically different for the HDPCM (7.20±3.08, p<0.001) and MFN (8.82±3.83, p<0.001) groups as compared to the SDPCM group (5.07±2.66). The mean plot comparing the means of the three groups is shown in Figure 4.

Adverse effects were also compared among the groups. Vomiting was observed in two (3.8%) patients in the SDPCM group and four (7.5%) in the MFN group. Dislikeness for food was noted in one (1.9%) each in the HDPCM and MFN groups. Daytime sleeping was reported in one (1.9%) each in the SDPCM and MFN groups. Among the study groups, there was no significant difference in adverse effects such as vomiting (χ2(2)=4.16, p=0.125), dislikeness for meals (χ2(2)=0.994, p=0.608), and daytime sleeping (χ2(2)=0.994, p=0.608) (Table 3).

**Table 3:** Adverse effects among groups n: number of patients, SDPCM: standard-dose paracetamol, HDPCM: high-dose paracetamol, MFN: mefenamic acid

Adverse effect	SDPCM group	HDPCM group	MFN group	χ2 value	p value
Vomiting (n (%))	2 (3.8)	0 (0)	4 (7.5)	4.160	0.125
Dislikeness for meals (n (%))	0 (0)	1 (1.9)	1 (1.9)	0.994	0.608
Daytime sleepiness (n (%))	1 (1.9)	0 (0)	1 (1.9)	0.994	0.608

Therefore, demographically, the participants were comparable in the three groups, and we found a statistically significant difference in the mean total time taken to reduce the temperature and the mean reduction of temperature in 60 minutes. Additionally, as per our hypothesis, it was found that the mean time taken for the reduction of temperature to normal was not significantly different in the high-dose paracetamol (20 mg/kg) and mefenamic acid groups, although the duration of action of mefenamic acid was longer than that of high-dose paracetamol.

## Discussion

Although paracetamol is the recommended antipyretic of choice for treating fever in children, NSAIDs are commonly prescribed in view of their rapid and prolonged duration of action, despite potential adverse effects [4]. Paracetamol is safely used even in higher doses [11]. We hypothesized that the antipyretic effect of paracetamol at a higher (20 mg/kg) dose would not be different from that of mefenamic acid.

In this study, demographically, the three groups were comparable. The mean time of the SDPCM group (97.50 minutes) was longer than that of the HDPCM (85.09 minutes) and MFN (84.90 minutes) groups. The difference in the mean reduction in temperature at 60 minutes was also statistically significant among the three groups. It was greater in the HDPCM (0.46°C) and MFN (0.45°C) groups than in the SDPCM (0.33°C) group. We found that the mean time of fall of temperature to normal and the mean fall of temperature in 60 minutes were comparable and statistically not significant in the HDPCM and MFN groups, suggesting that both are equivalent in antipyretic action. The time to the next fever spike was shorter for the SDPCM group (5.07 hours) than for the HDPCM (7.20 hours) and MFN (8.82 hours) groups. Although statistically not significant, the duration of action of mefenamic acid was longer than that of high-dose paracetamol, as suggested by the time of the next fever spike.

In other randomized trials, two or three groups were used to compare various drugs or combinations of drugs [12]. We included children in the age group of six months to five years. Other studies have enrolled children ranging from four months to 12 years. Wong et al. conducted a systematic review and included studies that enrolled children up to 18 years [3]. Kunkulol Rahul et al. included febrile children of one year to 12 years [13]. However, as age advances, dosage in mg/kg may not be linearly comparable and may sometimes exceed adult doses in obese children, e.g., if 15 mg/kg dose is used, a 10-year-old male with 50 kg weight will receive 750 mg of paracetamol.

As our main focus, we used paracetamol at a high dose (20 mg/kg). In other studies, paracetamol was safely used in higher doses for a short duration. Even a single dose of 30 mg/kg at bedtime was shown to increase the sleep time of the whole family [14]. A dose of 20 mg/kg was found to cause a larger and more prolonged decrease in temperature [11]. Tréluyer et al. randomly recruited 121 febrile children of four months to nine years into paracetamol 30 mg/kg and 15 mg/kg groups and found that “the time to obtain a temperature lower than 38.5°C was significantly shorter in the 30 mg/kg than in the 15 mg/kg group (110±94 minutes versus 139±113 minutes). The maximum temperature decrease was significantly higher in the 30 mg/kg than in the 15 mg/kg group (2.3°C±0.7°C versus 1.7°C±0.6°C). Duration of rectal temperature below 38.5°C was significantly longer in the 30 mg/kg than in the 15 mg/kg group (250±92 minutes versus 185±121 minutes, respectively)” [15]. Additionally, an initial dose of 40 mg/kg was also suggested to control postoperative pain in selected patients when used with precautions [16].

In this study, the axillary temperature was recorded in degrees Celsius. One study used tympanic temperature, although it may lead to erratic readings [17]. Most of the studies have used degree Celsius as the measure of temperature. Khubchandani et al. also used the degree Fahrenheit scale [6].

We measured the temperature every 15 minutes until the temperature reached normal (<37.22°C), although it is cumbersome for the caretaker. Other studies have used a frequency of measurement from 30 minutes [18] to one hour [13], to four hours [6]. Hourly and half-hourly measurements may not detect the time for the temperature to reach normal.

To study the antipyretic effect, we measured the mean time taken for reaching temperature to normal and the mean temperature to fall in 60 minutes and found a statistically significant difference between the groups, and the HDPCM and MFN groups had comparable antipyretic effects. Previous studies have some conflicting findings. Joshi et al., in a large multicentric trial, compared the antipyretic effects of ibuprofen (7 mg/kg) and paracetamol (8 mg/kg) and found both to be equally effective [19]. Kauffman et al. found that ibuprofen (7.5 mg/kg) provided a greater temperature decrement and longer duration of action than acetaminophen (10 mg/kg) [20]. Khubchandani et al. compared mefenamic acid (6.5 mg/kg), ibuprofen (7 mg/kg), and paracetamol (10 mg/kg) and found that mefenamic acid had a significantly better antipyretic effect than both and had a continued decrease in temperature even until four hours [6]. The dose of paracetamol was less than the standard recommended. Kunkulol Rahul et al. found that mefenamic acid had better antipyresis at one hour than acetaminophen [13]. Similar findings were also observed by Keinänen et al. [21], who also stated mefenamic acid to be a more potent and powerful antipyretic drug. Some other clinical trials found that 10 mg/kg of paracetamol was consistently less effective than ibuprofen [22,23]. Lal et al. randomly allocated 89 patients with temperatures above 38.5°C and administered nimesulide (1.5 mg/kg/dose), paracetamol (10 mg/kg/dose), or ibuprofen-paracetamol combination (10 mg/kg/dose) thrice daily for five days [24]. The ibuprofen-paracetamol combination, nimesulide, and paracetamol had almost similar antipyretic effects in children. From these studies, it is observed that all drugs have antipyretic effects, and differences may be due to different dosages, frequencies, or age groups of patients. A rough correlation has been established between the antisynthetase activities of many NSAIDs [22], including mefenamic acid, in the central nervous system.

We noted the time for the next fever spike and found that participants in the mefenamic acid group had a longer time for the next fever spike than the SDPCM group. Different diagnoses, the severity of illness, the day of illness, and other treatment measures may affect the appearance of the next fever spike. In the SDPCM group, most patients (54.7%) had the next fever spike within one to five hours. However, the maximum number of patients in the HDPCM group (45.3%) and the MFN group (58.5%) had the next fever spike in six to 10 hours.

We observed adverse effects clinically and found them negligible and comparable among groups. Only two patients in the SDPCM group and four in the MFN group had vomiting, one each in the SDPCM and HDPCM groups had dislikeness for meals, and one each in the SDPCM and MFN groups reported transient increased daytime sleep. The underlying illness may also have an effect on these complaints.

Our study has limitations. It was only single-centered, and blinding was not possible due to dissimilarity between drugs and their dose quantities. Only a single dose was used; hence, the outcomes of this study may not be similar for the long-term treatment, and there were no subgroups of intensities of fever that were measured in degrees Fahrenheit. We compared paracetamol with only mefenamic acid and not with other NSAIDs or combinations of drugs. Adverse effects were only clinically observed, and biochemical tests were not performed.

Further research is needed with a large sample size at multiple centers and comparing other NSAIDs or combinations. Studies may be carried out with the use of paracetamol throughout their entirety, and crossover trials may also help in further rigorous analysis.

## Conclusions

Standard-dose paracetamol (15 mg/kg) had slower and shorter antipyretic effects than high-dose paracetamol and mefenamic acid. The antipyretic effect of high-dose paracetamol (20 mg/kg) was as safe and effective as mefenamic acid when administered for the treatment of fever. The duration of action of mefenamic acid was prolonged, although it was not statistically significant. Therefore, we can choose paracetamol as a safe, first-line antipyretic in febrile children and use a higher dose (20 mg/kg) instead of mefenamic acid for equivalent results. Other NSAIDs may be spared for their anti-inflammatory indications or as a second-line antipyretic. Multicentered double-blind clinical trials with larger sample sizes and comparisons of other NSAIDs will be required to confirm these findings.
